# Endosome-to-Plasma Membrane Recycling of VEGFR2 Receptor Tyrosine Kinase Regulates Endothelial Function and Blood Vessel Formation

**DOI:** 10.3390/cells3020363

**Published:** 2014-04-29

**Authors:** Helen M. Jopling, Adam F. Odell, Caroline Pellet-Many, Antony M. Latham, Paul Frankel, Asipu Sivaprasadarao, John H. Walker, Ian C. Zachary, Sreenivasan Ponnambalam

**Affiliations:** 1 Endothelial Cell Biology Unit, School of Molecular & Cellular Biology, University of Leeds, Leeds LS2 9JT, UK; 2 Centre for Cardiovascular Biology and Medicine, The Rayne Institute, University College London, UK; 3 School of Biomedical Sciences, University of Leeds, Leeds LS2 9JT, UK

**Keywords:** endothelial, VEGF-A, VEGFR2, Rab4a, Rab11a, signalling, angiogenesis

## Abstract

Rab GTPases are implicated in endosome-to-plasma membrane recycling, but how such membrane traffic regulators control vascular endothelial growth factor receptor 2 (VEGFR2/KDR) dynamics and function are not well understood. Here, we evaluated two different recycling Rab GTPases, Rab4a and Rab11a, in regulating endothelial VEGFR2 trafficking and signalling with implications for endothelial cell migration, proliferation and angiogenesis. In primary endothelial cells, VEGFR2 displays co-localisation with Rab4a, but not Rab11a GTPase, on early endosomes. Expression of a guanosine diphosphate (GDP)-bound Rab4a S22N mutant caused increased VEGFR2 accumulation in endosomes. TfR and VEGFR2 exhibited differences in endosome-to-plasma membrane recycling in the presence of chloroquine. Depletion of Rab4a, but not Rab11a, levels stimulated VEGF-A-dependent intracellular signalling. However, depletion of either Rab4a or Rab11a levels inhibited VEGF-A-stimulated endothelial cell migration. Interestingly, depletion of Rab4a levels stimulated VEGF-A-regulated endothelial cell proliferation. Rab4a and Rab11a were also both required for endothelial tubulogenesis. Evaluation of a transgenic zebrafish model showed that both Rab4 and Rab11a are functionally required for blood vessel formation and animal viability. Rab-dependent endosome-to-plasma membrane recycling of VEGFR2 is important for intracellular signalling, cell migration and proliferation during angiogenesis.

## 1. Introduction

Membrane receptor recycling is an important function in regulating animal physiology [1,2,3]. Although the machinery controlling recycling from intracellular endosomes has not been fully characterised, endosome-associated Ras-related (Rab) GTPases are implicated in regulating such pathways [4]. Two such Rab GTPases, Rab4a and Rab11a, regulate different recycling routes from early endosomes back to the plasma membrane [5]. In endothelial cells, Rab4a is functionally involved in membrane protein recycling from endosomes, exemplified by vascular endothelial growth factor receptors (VEGFRs) and αVβ3 integrin [5]. A Rab-independent and c-Src-dependent pathway has been postulated to regulate recycling from endosome-to-plasma membrane in endothelial cells [6].

The vascular endothelial growth factor (VEGF) family of cytokines plays essential roles in vasculogenesis, angiogenesis and animal physiology [2,7,8]. Vascular endothelial growth factor A (VEGF-A) is the most well-characterized family member. Different splice variants exist with VEGF-A_165_ (termed VEGF-A) being the most abundant and well-studied family member [9]. Loss of a single VEGF-A allele impairs vascular development and causes embryonic lethality, emphasizing the functional importance of VEGF-A in animal physiology [10,11]. VEGF-A can bind to two receptor tyrosine kinases (RTKs), *i.e.*, either fms-like tyrosine kinase (VEGFR1 or Flt-1) or kinase insert domain receptor (VEGFR2 or KDR). VEGF-A binding to RTKs can also recruit membrane co-receptors, called neuropilins (NRP1, NRP2). VEGF-A-stimulated VEGFR2 activation in endothelial cells triggers multiple signal transduction steps, such as extracellular signal-regulated kinase (ERK1/2), the serine/threonine protein kinase Akt (PKB) and endothelial nitric oxide synthase (eNOS) *inter alia* [7]. Such pro-angiogenic signal transduction cascades regulates endothelial functions ranging from cell survival, proliferation, migration, tubulogenesis and angiogenesis to vasculogenesis [8].

VEGFR2-regulated signal transduction events have been intensively studied, but how this is coordinated with receptor trafficking and vascular physiology is poorly understood. Whilst endocytosis of RTKs can attenuate signalling events, such outputs can differ, dependent on the location within the endocytic pathway [12,13,14]. Activated RTKs usually have two possible fates: recycling back to the plasma membrane or degradation within the endosome-lysosome pathway. At steady state, quiescent VEGFR2 is localised to both the plasma membrane and early endosomes [6,12,15]; ligand-stimulated activation causes VEGFR2 trans-autophosphorylation, ubiquitination, endosome and lysosome-linked proteolysis [14]. Both quiescent and activated VEGFR2 can be recycled [6,15], but how this is balanced with lysosomal delivery for proteolysis is not understood. Here, we test a role for Rab GTPases that regulate different endosome-to-plasma membrane routes. These studies reveal that VEGFR2 exhibits unique dependence on Rab4a and Rab11a activity in controlling endothelial function, vascular development and physiology.

## 2. Experimental Section

### 2.1. Materials, Cell Culture, Microscopy and Flow Cytometry

Recombinant human VEGF-A_165_ was a gift from Genentech Inc. (San Francisco, CA, USA). Isolation and culture of primary human umbilical vein endothelial cells (HUVECs) was described previously [16]. Purified goat anti-VEGFR2 extracellular domain (R&D Systems, Abingdon, UK) and mouse monoclonal anti-Rab4a antibodies (BD Biosciences, Oxford, UK) were used with horseradiah peroxidase (HRP)-conjugated secondary antibodies (ThermoFisher, Loughborough, UK) and AlexaFluor-conjugated secondary antibodies (Invitrogen, Amsterdam, Netherlands). Non-endothelial cell culture medium and supplements were from Invitrogen (Paisley, UK), whereas endothelial cell growth medium and supplements were from Promocell (Heidelberg, Germany). HUVECs were fixed and processed for immunofluorescence microscopy, as described previously [16,17]. All other reagents were purchased from Sigma-Aldrich (Poole, UK), unless otherwise stated. 

For flow cytometry [17], HUVECs were treated as appropriate, and medium was removed from cells and kept on ice. Cells were trypsinized and resuspended in original media. Cells were rinsed in ice-cold phosphate-buffered saline (PBS) and fixed in 3% paraformaldehyde for 20 min. After washes in blocking buffer (1 mg/mL bovine serum albumin (BSA) in PBS), cells were incubated with goat anti-VEGFR2 (10 μg/mL) for 30 min, washed three times and then incubated with rabbit anti-goat AlexaFluor488 conjugate (10 μg/mL) for 30 min. Cells were washed twice more in binding buffer followed by the addition of 2 mg/mL of 4',6'-diamidino-2-phenylindole (DAPI) prior to analysis using a Fortessa flow cytometer (Beckton Dickinson, U.K.). Cells labelled with DAPI alone were used as controls to set up appropriate gating parameters.

Cycloheximide (CHX) was routinely used to inhibit new protein synthesis and deplete Golgi and ER-associated VEGFR2 and monitor only the plasma membrane and endosomal pools of VEGFR2. CHX (50 μg/mL) was used for 2 h during the VEGF-A stimulation period before fixation or cell lysis for further analysis.

### 2.2. Gene Manipulation and RNA Interference

Cells were transfected with GFP-tagged human Rab4a (Francis Barr, University of Oxford, UK), human Rab5a (Brian Knoll, University of Texas, USA) or canine Rab11a (Nigel Bunnett, Monash University, Australia) wild-type or mutant proteins, as previously described [17]. HUVECs were transfected with siRNA duplexes using Lipofectamine 2000 as specified (Invitrogen, Amsterdam, Holland). Cells were assayed 48 h following transfection. siRNA duplexes targeting human Rab4a and Rab11a were designed, synthesized and annealed. RNA interference (RNAi) targeting Rab4a had a sense sequence of 5' GUUCUUGGUUAUUGGAAAU 3'. Non-targeting control siRNA duplex (Silencer Negative Control #1; Ambion, Warrington, U.K.) was also used.

### 2.3. Intracellular Signalling Analysis

HUVEC lysate preparation and immunoblotting were performed as described previously [12,14,17]. Briefly, confluent HUVEC monolayers were lysed in 2% (w/v) SDS in PBS and the lysate subsequently boiled for 5 min at 95 °C. Proteins were separated by SDS-PAGE on 10% gels and then transferred onto nitrocellulose membranes. The following antibodies were used: anti-VEGFR2 extracellular domain (R&D Systems, Minneapolis, USA), anti-Akt, anti-phospho-Akt (pS473), anti-ERK1/2, anti-phospho-ERK1/2, anti-phospho-VEGFR2 (pY1175), anti-phospho-p38 (Cell Signaling Technology, Danvers, USA), anti-Rab4a, anti-Rab11a and anti-α-tubulin (Santa Cruz Biotechnology, Santa Cruz, USA). Immunoreactive bands were visualized using an enhanced chemiluminescence detection kit (Geneflow, Nottingham, U.K.). Antibodies to α-tubulin were used as internal controls in immunoblot experiments.

### 2.4. Cell Migration, Proliferation and Tubulogenesis Assays

Endothelial cell migration assays were carried out as described previously [17,18,19]. Control or siRNA-treated confluent HUVEC monolayers were trypsinised and seeded at 5 × 10^4^ cells/mL into a 24-well plate with 8-µm pore size Transwell inserts (BD Biosciences, Oxford, UK). Endothelial cell migration was stimulated by VEGF-A (50 ng/mL) in the lower chamber. After 16 h, the Transwell units were fixed in 3% paraformaldehyde, stained with hematoxylin-eosin and circular inserts excised for microscopy analysis. Digital microscopy images of the underside (containing migratory endothelial cells) of each Transwell insert were analysed; random fields from each image dataset were evaluated for the percentage (%) of migrated endothelial cells. In the cell proliferation assay [17], endothelial cells were subjected to control (scrambled), Rab4a or Rab11a siRNA treatment for the specified period, trypsinized and seeded at 1,000 cells per well in 96-well plates and grown for 24 h and processed using a 5-bromo-2-deoxyuridine (BrdU) ELISA (Roche Diagnostics, Lewes, UK). An *in vitro* endothelial tubulogenesis assay involved the co-culture of endothelial cells on a primary fibroblast monolayer. Endothelial cells were subjected to control (scrambled), Rab4a or Rab11a siRNA treatment for the specified period, trypsinised and seeded on a bed of human primary fibroblasts in 24-well plates and cultured for 7 days in growth medium containing VEGF-A (50 ng/mL). Cells were fixed using paraformaldehyde, stained and imaged by phase contrast microscopy as previously described [17]. Tubule length was quantified using Image J (NIH, Bethesda, MD, USA) software.

### 2.5. Transgenic Zebrafish Manipulation and Analysis

This was carried out as previously described [20]. Four nanolitres of morpholino (Gene-Tools, Philomath, USA) in a 100 μM stock solution were injected into the yolk sac of transgenic *Fli1-GFP* embryos at the 1- to 4-cell stage. In each treatment, ~100 embryos were injected and allowed to develop for 48 h. The injected morpholino sequences were: *Rab4a*: 5' CAAGAAATCGTATGTCTCTGACAT 3'; *Rab11a:* 5' GTATTCGTCGTCTCGTGTCCCCATC 3'. Pictures of the zebrafish embryos were obtained under brightfield using a NikonSMZ1500 optical microscope. Microscopy data were acquired using a Leica TCS SP2 confocal microscope (excitation at 488 nm) at a 100× magnification. To check for Rab knockdown, 100 dechorionated embryos were washed twice in cold Ringer’s solution (116 mM NaCl, 2.9 mM KCl, 1.8 mM CaCl_2_, 5 mM Hepes, pH 7.2), followed by mechanical removal of the yolk sac through a thin pipette tip. Embryos were lysed, sonicated in lysisuffer (30 mM Tris-HCl pH 7.4, 150 mM NaCl, 1% (v/v) NP-40, 0.5% (w/v) deoxycholate, 2 mM ETDA) and SDS sample buffer added. SDS-PAGE and immunoblotting using primary and HRP conjugated secondary antibodies were carried out as previously described.

### 2.6. Statistical Analysis

Datasets and error bars denote the mean ± SEM (standard error of the mean). The statistical significance of differences between datasets was analysed using the Student’s *t*-test, where *p* < 0.05 was considered significant.

## 3. Results and Discussion

### 3.1. VEGFR2 Recycles to the Plasma Membrane via Rab4a-Containing Endosomes

**Figure 1:**
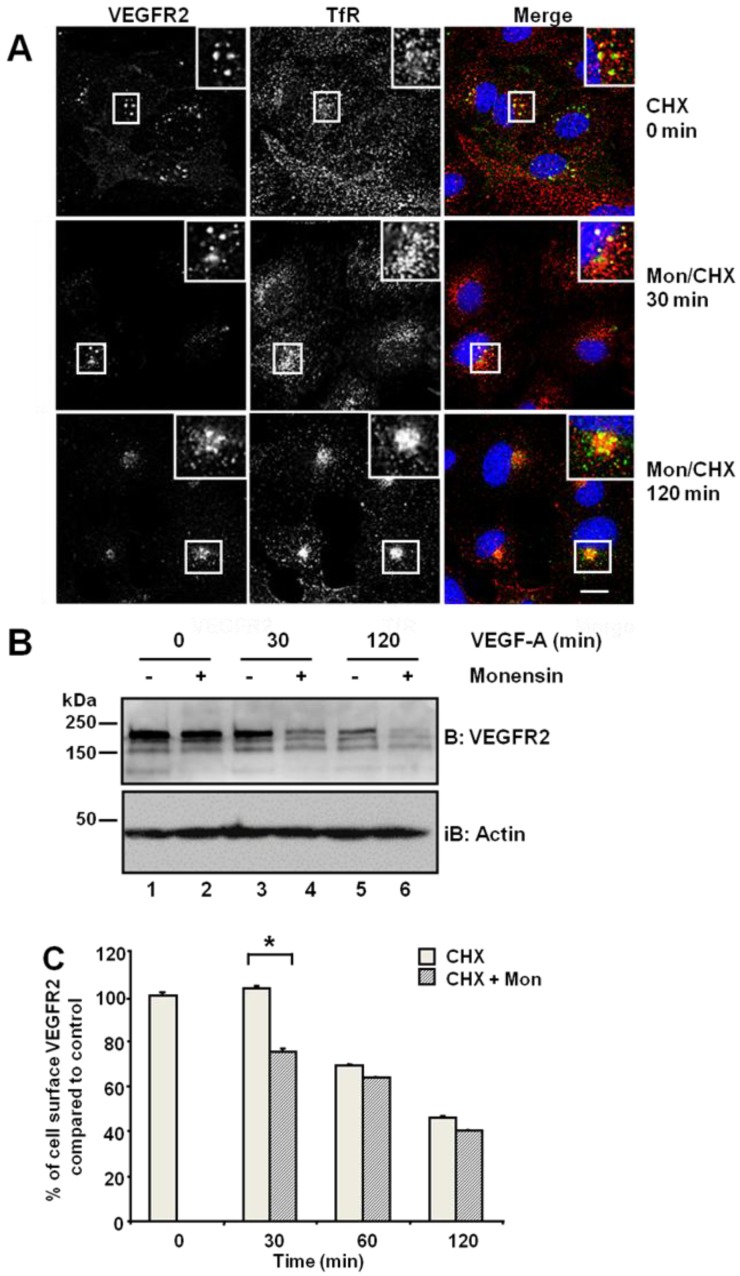
Inhibition of endosome-to-plasma membrane recycling restricts VEGFR2 to a perinuclear compartment. (**A**) Human umbilical vein endothelial cells (HUVECs) were pre-treated for 2 h with cycloheximide (CHX) prior to treatment with 20 μM monensin for zero, 30 or 120 min. Cells were then fixed, permeabilised and labelled with goat anti-VEGFR2 extracellular domain (green) and mouse anti-TfR (red). Primary antibodies were visualized using AlexaFluor-conjugated species-specific secondary antibodies, and the nucleus was labelled with DAPI (blue). Bar: 10 μm. Insets show a two-fold magnification of the indicated regions. (**B**) Serum-starved HUVECs were pre-treated with 20 μM monensin prior to VEGF-A stimulation for zero, 30 or 120 min. Cell lysates were immunoblotted with antibodies specific for VEGFR2 or actin. The data shown is representative of three independent experiments. (**C**) Flow cytometry analysis of HUVECs treated with either 20 μM monensin or 50 μg/mL cycloheximide (CHX) for zero, 30, 60 or 120 min. Error bars denote ± SEM (n = 3); *, *p* < 0.05.

Previous work has shown that the VEGFR2 exhibits both plasma membrane and endosomal localisation [6,12,15,21]. To test whether VEGFR2 undergoes a ligand-independent endosome-to-plasma membrane recycling step in quiescent primary human endothelial cells, monensin can be used, as this agent inhibits cell surface receptor recycling [22,23,24,25]. Primary endothelial cells were first subjected to a block in new protein synthesis using cycloheximide: we detected a stable and distal pool of VEGFR2, which showed partial co-distribution with the transferrin receptor (TfR) present in early endosomes (Figure 1A, upper panels). Simultaneous treatment with monensin and cycloheximide for 30 min increased VEGFR2 co-distribution with TfR in early endosomes (Figure 1A, insets). Following simultaneous VEGF-A and cycloheximide treatment, VEGFR2 levels markedly decreased in the absence of new protein synthesis (Figure 1B). Monensin further potentiated this VEGF-A-stimulated decrease in VEGFR2 levels (Figure 1B). Inhibition of endosome-to-plasma membrane recycling would be thus predicted to decrease cell surface VEGFR2 levels. Flow cytometry analysis showed a ~25% decrease in cell surface VEGFR2 levels after 30-min exposure to monensin, compared with cycloheximide-treated cells, which showed no significant change in VEGFR2 levels (Figure 1C). Thus, both quiescent and activated VEGFR2 undergoes endosome-to-plasma membrane recycling.

**Figure 2:**
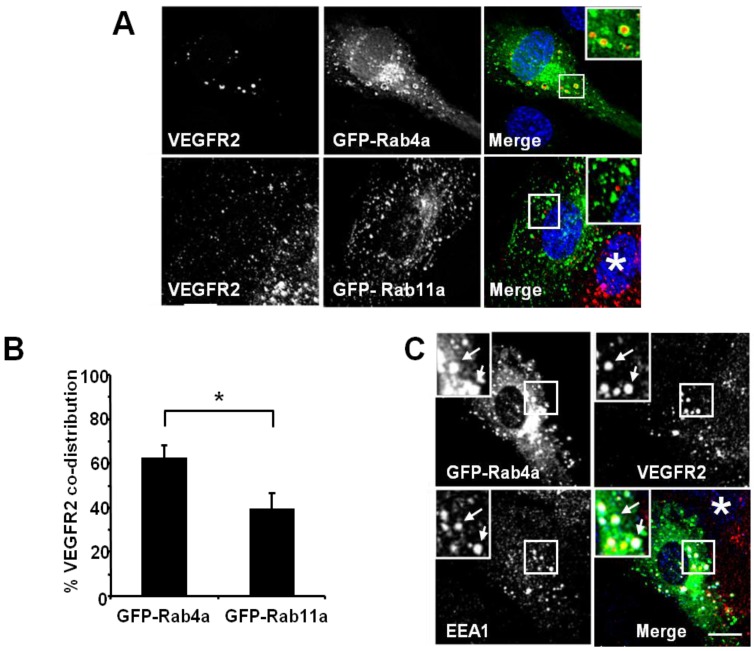
VEGFR2 trafficking in early endosomes is closely associated with the Rab4a GTPase. (**A**) HUVECs were transfected with either GFP-Rab4a or GFP-Rab11a (green), fixed and probed with goat antibodies to the VEGFR2 extracellular domain, followed by AlexaFluor-labelled secondary antibodies (red). The nucleus was visualised with DAPI (blue). (**B**) Quantification of VEGFR2 co-localisation with GFP-Rab4a or GFP-Rab11a (see Materials and Methods). Error bars denote SEM (n = 45); * *p* < 0.05. (**C**) VEGFR2 is present in Rab4a-positive early endosomes. HUVECs were transiently transfected to express GFP-Rab4a (green), and cells were fixed, permeabilised and labelled with goat anti-VEGFR2 (red) and rabbit anti-EEA1 (blue). Bound primary antibodies were visualised with AlexaFluor-conjugated secondary antibodies. Insets show a two-fold magnification of the highlighted region. Bar: 10 μm.

Rab GTPases, such as Rab4a or Rab11a, regulate different endosome-to-plasma membrane recycling routes [26,27,28,29,30,31,32] and are functionally linked to VEGFRs in endothelial cells [6,21,33]. To differentiate between these two endosome-linked recycling pathways, we overexpressed GFP-tagged wild-type Rab4a or Rab11a in endothelial cells and assessed co-distribution with VEGFR2 using quantitative microscopy (Figure 2). Endothelial cell transfection and expression of GFP-Rab4a caused the accumulation of punctate structures with Rab4a-enriched membranes and a central core of ‘trapped’ VEGFR2 within the internal lumen (Figure 2A, upper panels and inset). In contrast, transfection and expression of GFP-Rab11a showed non-overlapping distribution of VEGFR2 and Rab11a to different vesicles (Figure 2A, lower panels and inset). Quantification of VEGFR2 co-distribution of either Rab4a or Rab11a in such experiments revealed a significant difference between these two GTPases (Figure 2B). To further assess the link between early endosomes and VEGFR2 dynamics, we compared the co-distribution of early endosomal antigen 1 (EEA1), GFP-Rab4a and VEGFR2 (Figure 2C). These experiments showed that a proportion of EEA1-positive endosomes also contained Rab4a and VEGFR2 (Figure 2C, arrows).

### 3.2. VEGFR2 Endosome-to-Plasma Membrane Recycling Depends on Rab4a GTPase Activity

**Figure 3:**
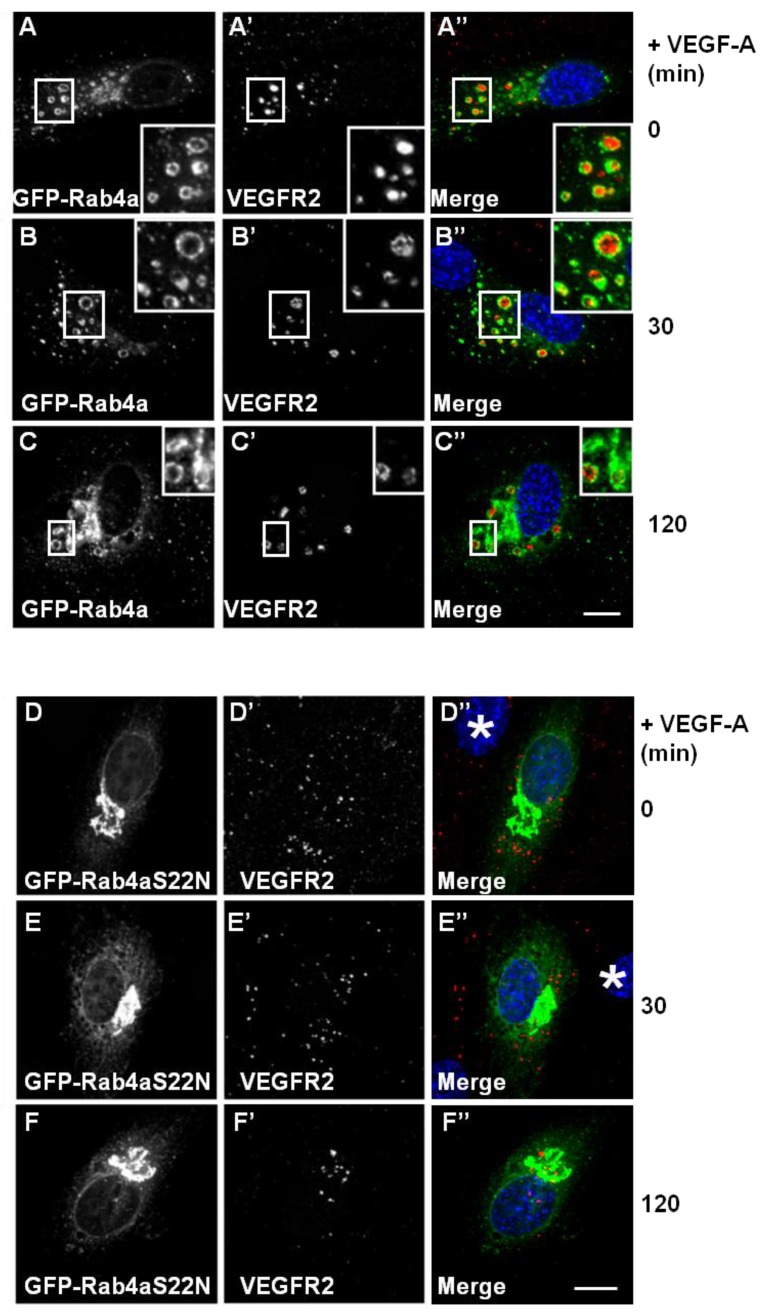
Wild-type and mutant Rab4a-S22N blocks VEGF-A-stimulated VEGFR2 degradation. (**A**–**C**) HUVECs were transfected with GFP-Rab4a (green) and cells were then stimulated with VEGF-A for (**A**) 0 min, (**B**) 30 min or (**C**) 120 min in the presence of CHX. Cells were subsequently fixed, permeabilised and labelled with goat anti-VEGFR2 followed by AlexaFluor-conjugated secondary antibody (red). The nucleus is visualized using DAPI (blue). Inset panels show a two-fold magnification of boxed highlighted regions. Bar: 10 μm. (**D**–**F**) HUVECs were transiently transfected to express dominant-negative GDP-bound GFP-Rab4a-S22N (green) and then stimulated with VEGF-A for (**D**) 0 min, € 30 min or (**F**) 120 min in the presence of cycloheximide (CHX) and processed for immunofluorescence microscopy. VEGFR2 was detected using goat anti-VEGFR2 followed by AlexaFluor-conjugated secondary antibody (red), whilst the nuclear DNA was labelled with DAPI (blue). The images shown are representative of three independent experiments. Inset panels show a two-fold magnification of boxed highlighted regions. Bar: 10 μm.

VEGF-A-activated VEGFR2 undergoes ubiquitination and proteolysis within the endosome-lysosome system within 2 h, even when new protein synthesis is blocked [12]. To address whether Rab4a influences VEGFR2 trafficking and degradation, we analysed VEGF-A-stimulated VEGFR2 activation and internalisation in GFP-Rab4a-expressing endothelial cells (Figure 3A–C). VEGFR2-positive/Rab4a-positive endosomes were prominent even 2 h after ligand stimulation in the presence of cycloheximide to inhibit further protein synthesis (Figure 3C’, insets). This delayed VEGFR2 trafficking effect was linked to increased Rab4a expression; and therefore, may be regulated by the Rab4a guanosine triphosphate **(**GTP)/guanosine diphosphate (GDP)-bound state. To test this, we overexpressed a GDP-bound dominant-negative Rab4a mutant (GFP-Rab4a-S22N) in endothelial cells and evaluated VEGFR2 trafficking and distribution following VEGF-A stimulation using microscopy (Figure 3D–F). This GDP-bound Rab4a mutant may be expected to prevent the recycling of VEGFR2 and, therefore, enhances the proportion of the receptor pool that is subject to lysosomal degradation. GDP-bound Rab4a-S22N localised to a juxtanuclear membrane compartment clearly distinct from VEGFR2 localization (Figure 3D). Endothelial cells expressing juxtanuclear Rab4a-S22N did not show co-distribution with endosomal VEGFR2; however, in these cells, endosomal VEGFR2 levels persisted (Figure 3D’–F’). Interestingly, VEGF-A-stimulation still exhibited increased VEGFR2 retention within endosomes and reduced VEGFR2 degradation (Figure 3F’) in comparison to control non-transfected cells.

**Figure 4:**
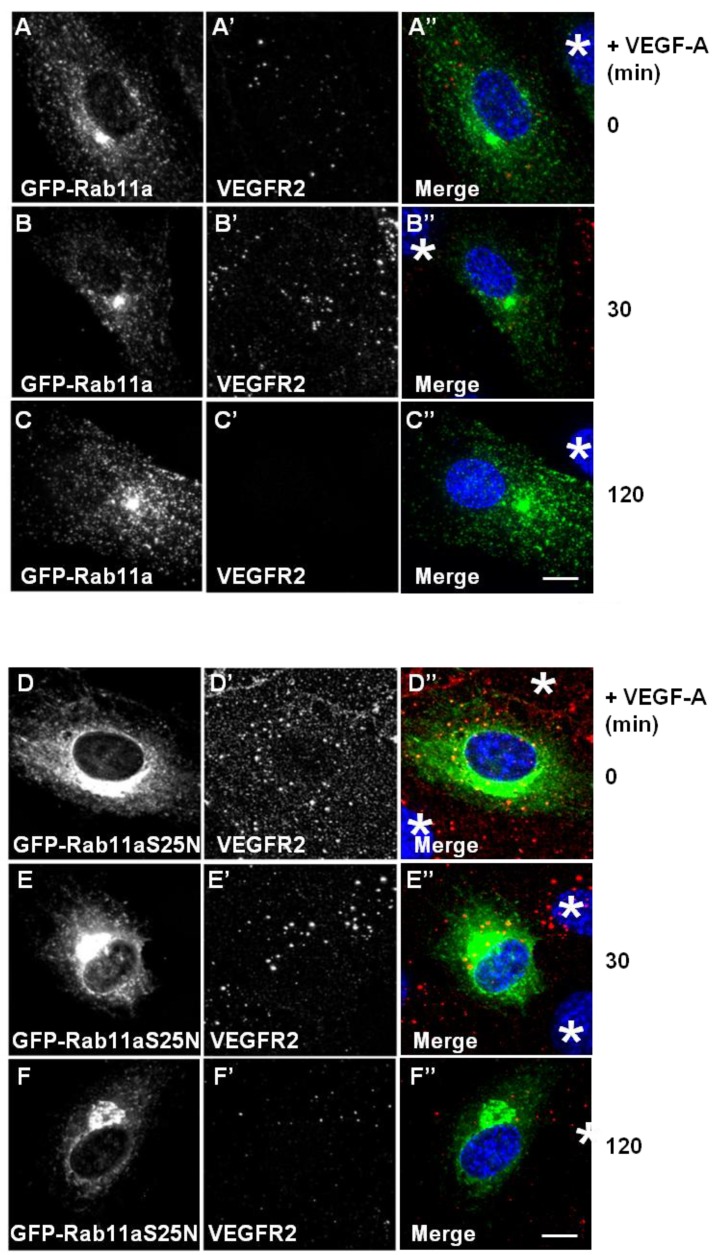
Rab11a perturbation does not affect VEGF-A stimulated VEGFR2 degradation. (**A**–**C**) HUVECs were transiently transfected to express GFP-Rab11a (green) and then stimulated with VEGF-A for (**A**) 0 min, (**B**) 30 min or (**C**) 120 min in the presence of cycloheximide (CHX) and processed for immunofluorescence microscopy. VEGFR2 was detected using goat anti-VEGFR2 antibody followed by AlexaFluor-conjugated secondary antibody (red), whilst the nuclear DNA was labelled with DAPI (blue). The images shown are representative of three independent experiments. Inset panels show a two-fold magnification of boxed highlighted regions. Bar: 10 μm. (**D**–**F**) HUVECs were transiently transfected to express dominant-negative GDP-bound GFP-Rab11a-S25N (green) and then stimulated with VEGF-A for (**D**) 0 min, (**E**) 30 min or (**F**) 120 min in the presence of cycloheximide (CHX) and processed for immunofluorescence microscopy. VEGFR2 was detected using goat anti-VEGFR2 followed by AlexaFluor-conjugated secondary antibody (red), whilst the nuclear DNA was labelled with DAPI (blue). The images shown are representative of three independent experiments. Inset panels show a two-fold magnification of boxed highlighted regions. Bar: 10 μm.

Different membrane receptors recycle from the endosome-to-plasma membrane via Rab4a- or Rab11a-dependent pathways [26,29]. To further test for Rab11a involvement in the regulation of VEGFR2 endosome-to-plasma membrane recycling, we overexpressed either wild-type or dominant-negative (S25N) Rab11a proteins in endothelial cells and stimulated with VEGF-A (Figure 4). Expression of GFP-Rab11a in endothelial cells followed by VEGF-A stimulation revealed two findings. Firstly, there was little or no co-distribution between VEGFR2 and Rab11a (Figure 4A,B). Secondly, VEGF-A-stimulated VEGFR2 activation for 2 h caused noticeable VEGFR2 degradation (Figure 4C’) in cells overexpressing Rab11a (Figure 4C), thus resembling control non-transfected endothelial cells (see Figure 1A). We also overexpressed a GDP-bound dominant-negative Rab11a mutant (GFP-Rab11a-S25N) and evaluated VEGFR2 trafficking and distribution in transfected endothelial cells (Figure 4D–F). Interestingly, GDP-bound Rab11a-S25N also localised to a juxtanuclear membrane compartment in transfected endothelial cells (Figure 4D), and this was distinct from VEGFR2 distribution (Figure 4D’). In contrast to previous experiments with Rab4a-S22N, Rab11a-S25N expression did not significantly affect VEGF-A-stimulated VEGFR2 activation and degradation via the endosome-lysosome system (Figure 4F’).

### 3.3. The VEGFR2 and TfR Recycling Pathways Are Distinct and Modulate Signal Transduction

**Figure 5:**
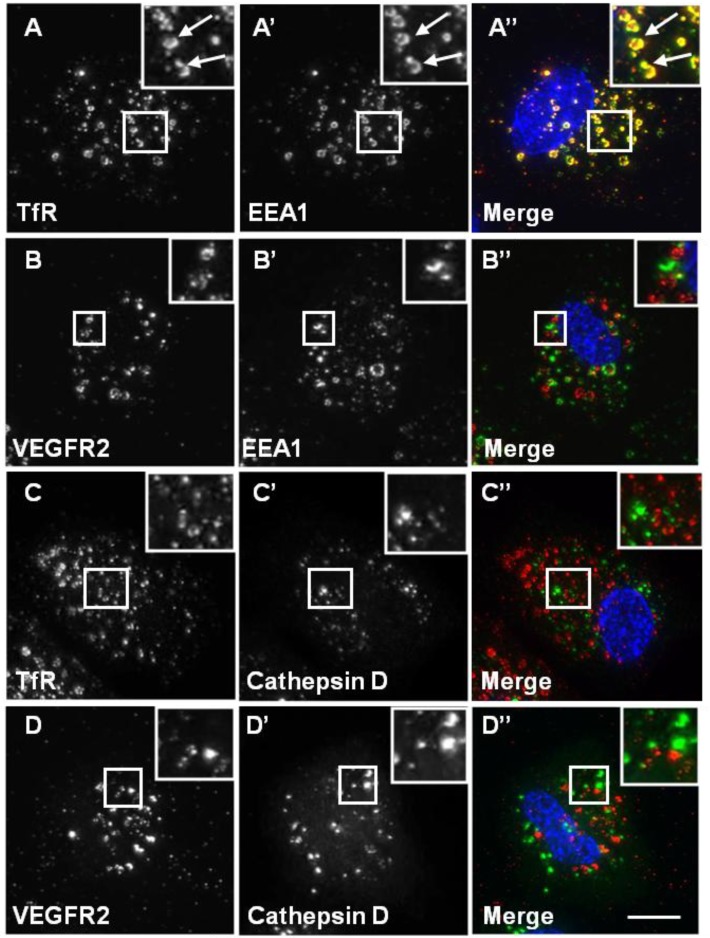
Chloroquine causes TfR, but not VEGFR2, accumulation in endosomes. A direct recycling assay was performed using the goat anti-VEGFR2 extracellular domain or mouse anti-TfR (red). Samples were fixed, permeabilised and labelled with either rabbit anti-EEA1 (green) or rabbit anti-cathepsin D (green). Primary antibodies were visualised using either FITC anti-rabbit IgG or AlexaFluor-488-conjugated anti-rabbit IgG, and the nucleus was seen with DAPI (blue). The images are 2D projections of a stacked series of 15–30 μm optical sections taken using a wide field deconvolution microscopy system. Insets show a two-fold magnification of the indicated region. Bar: 10 μm.

A number of previous studies have established that VEGFR2 undergoes endosome-to-plasma membrane recycling [6,15,21]. We used the previously described antibody-based recycling assay to monitor VEGFR2 and TfR recycling [15]. Chloroquine (CHQ) is an inhibitor of endosomal compartment acidification and, therefore, blocks endocytosis [34]. Upon CHQ treatment of endothelial cells, both recycling VEGFR2 and TfR accumulated in enlarged endosomal structures (Figure 5). Intriguingly, whilst TfR showed a high level of co-localisation with EEA1 (Figure 5A), VEGFR2 showed little co-localisation with EEA1 under similar conditions (Figure 5B). These data indicate differences in VEGFR2 and TfR recycling. Neither VEGFR2 nor TfR showed any co-localisation with cathepsin D in the presence of CHQ (Figure 5C and D).

Does Rab4a GTPase activity and regulation of endosome-to-plasma membrane recycling influence VEGF-A-stimulated intracellular signalling in endothelial cells? We have previously used RNA interference (RNAi) to evaluate Rab5a and Rab7a GTPase regulation of VEGFR2 function linked to endosome-lysosome trafficking and VEGF-A-stimulated intracellular signalling in primary endothelial cells [17]. We used this approach to check whether Rab4a or Rab11a depletion modulated VEGF-A-stimulated short-term intracellular signalling, therefore altering longer term cellular responses, such as endothelial cell migration, proliferation and endothelial tube formation, *i.e.*, tubulogenesis. 

**Figure 6:**
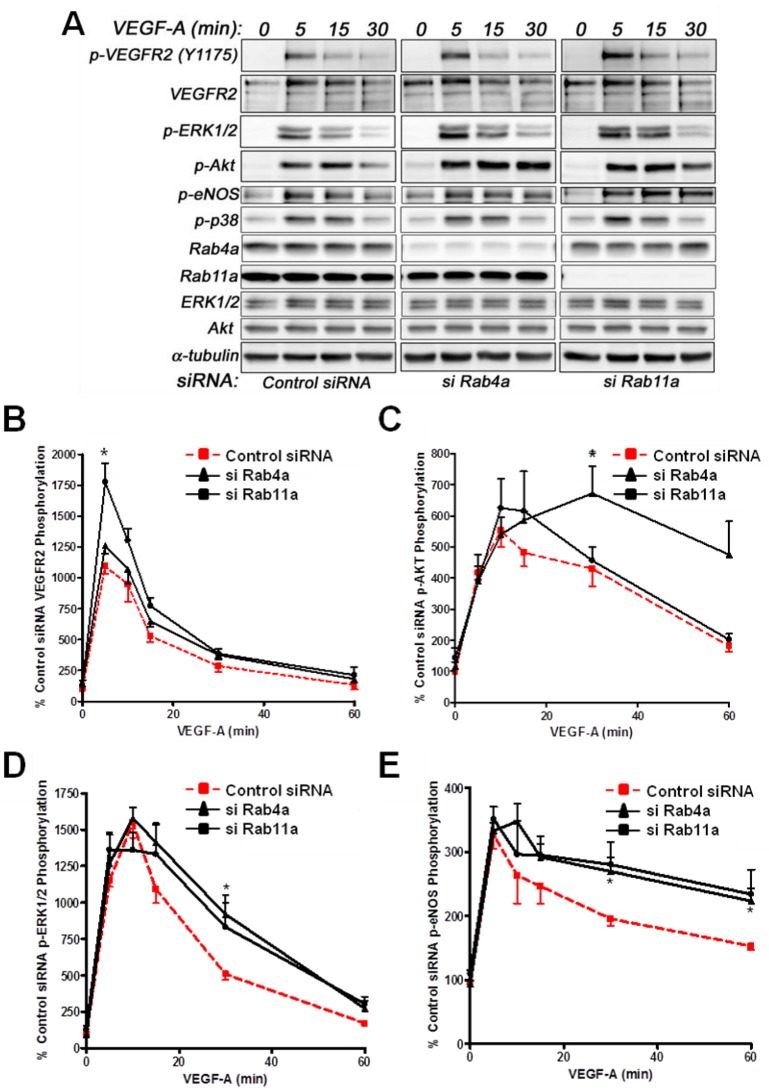
Effects of Rab silencing on VEGF-A-stimulated intracellular signalling in endothelial cells. (**A**) HUVECs were subjected to RNAi (see Materials and Methods) and the control; Rab4a- or Rab11a-depleted HUVECs were then stimulated with VEGF-A followed by quantitative immunoblotting for (**B**) VEGFR2-pY1175, (**C**) phosphorylated Akt (p-Akt S473), (**D**) phosphorylated MAPK (p42/44 MAPK or p-ERK1/2 T202/Y204) and (**E**) phosphorylated eNOS (p-eNOS S1179). The data shown are derived from the analysis of three or more independent experiments. Error bars denote ±SEM, with asterisks indicating significance (*, *p* < 0.05).

Analysis of VEGF-A-stimulated VEGFR2 activation revealed subtle differences in VEGFR2 activation and downstream signal transduction (Figure 6A). Quantification showed a nearly two-fold increase in VEGFR2-pY1175 levels upon Rab11a depletion, whereas Rab4a depletion did not significantly affect VEGFR2-pY1175 levels (Figure 6B). Furthermore, VEGF-A-stimulated Akt activation was prolonged upon Rab4a depletion compared to controls and Rab11a-depleted cells, although peak phospho-Akt levels were not significantly altered (Figure 6C). However, the effects on p42/44 MAPK (ERK1/2) activation were less pronounced (Figure 6D). Rab4a or Rab11a depletion did not affect peak phospho-p42/44 MAPK levels, but the duration of this species was prolonged (Figure 6D). Similarly, although Rab4a and Rab11a depletion did not affect peak levels of VEGF-A-stimulated intracellular signalling and activation of endothelial nitric oxide synthase (eNOS), the duration of the activated phospho-eNOS (peNOS) species was also prolonged; an affect more pronounced upon Rab11a depletion (Figure 6E). The activation of p38 MAPK was unaffected by the depletion of either Rab4a or Rab11a. 

### 3.4. Rab GTPase Regulation of Intracellular Signalling Cell Migration, Proliferation and Tubulogenesis

**Figure 7:**
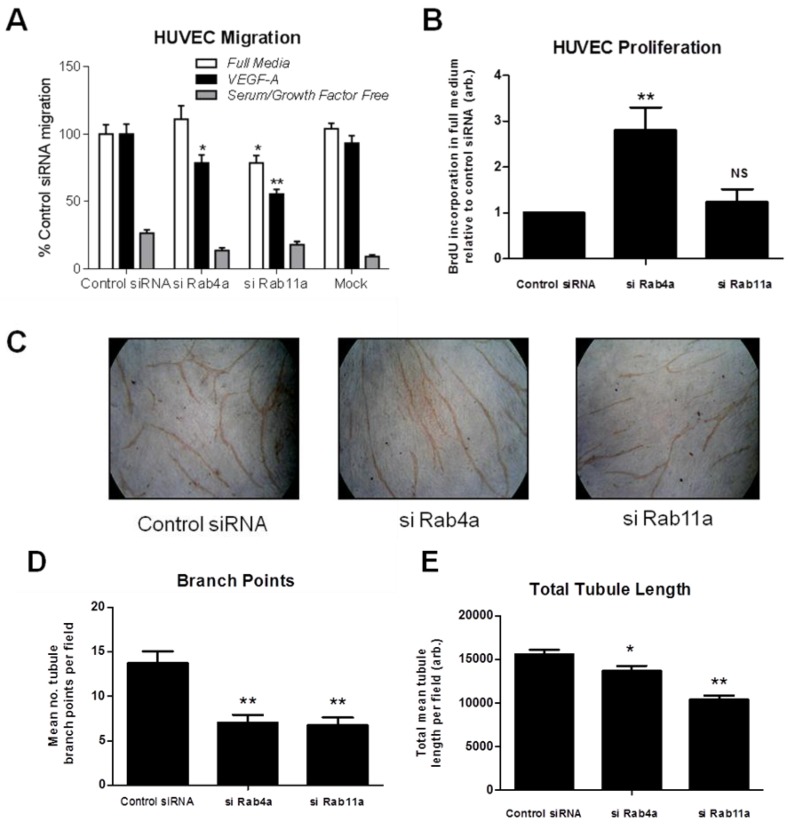
Rab-mediated regulation of endothelial cell migration, proliferation and *in vitro* angiogenesis. HUVECs were subjected to RNAi (see Materials and Methods) and the control; Rab4a- or Rab11a-depleted HUVECs were then analysed for (**A**) cell migration using a Transwell assay in complete media, VEGF-A-supplemented minimal media or in minimal media alone, (**B**) endothelial cell proliferation using a 5-bromo-2-deoxyuridine (BrdU)-modified nucleotide DNA incorporation ELISA or (**C**) endothelial tubule formation (tubulogenesis) using an organotypic endothelial-fibroblast co-culture assay. RNAi-activated HUVECs were trypsinized and plated on a bed of confluent human fibroblasts and allowed to grow in the presence of excess VEGF-A for seven days before endothelial tubules became visible. Co-cultures were fixed, stained with anti-PECAM-1 (CD31) antibody and HRP-conjugated secondary antibody followed by processing for light microscopy. Endothelial tubules were then analysed for (**D**) the number of branch points and (**E**) the total tubule length after tubulogenesis using Image J software. Error bars denote ±SEM; *, *p* < 0.05; **, *p* < 0.01.

To test if altered Rab-dependent VEGFR2 recycling influences VEGF-A responses, we analysed endothelial cell migration in Rab4a or Rab11a-depleted endothelial cells (Figure 7A). Endothelial cells subjected to Rab4a knockdown showed ~20% reduction in cell migration in VEGF-A-supplemented minimal medium compared to complete medium (Figure 7A). In cells subjected to Rab11a knockdown, cell migration was reduced ~20% in complete media and reduced ~40% in VEGF-A-supplemented minimal media (Figure 7A). To assess such effects for another physiological response, such as VEGF-A-stimulated cell proliferation, endothelial cells subjected to Rab4a knockdown showed nearly a three-fold increase in cell proliferation relative to controls (Figure 7B). In contrast, Rab11a-depleted endothelial cells did not show altered cell proliferation (Figure 7B). In the absence of VEGF-A or adequate medium for >6–12 h, there is pronounced endothelial necrosis/apoptosis that is further accentuated by siRNA treatments. To control for this aspect, we used comparisons with scrambled siRNA controls (control siRNA) and other Rab-specific siRNAs as described here.

Next, we used an organotypic endothelial-fibroblast co-culture assay to recapitulate the endothelial tube formation characteristic of VEGF-A-stimulated angiogenesis. We analysed endothelial tubulogenesis in Rab4a or Rab11a-depleted endothelial cells subjected to sustained VEGF-A treatment for seven days (Figure 7C). Endothelial cells showed ~50% reduction in the incidence of branch points in tubular networks upon depletion of either Rab4a or Rab11a GTPase (Figure 7C and D). Interestingly, VEGF-A-stimulated endothelial tubule length was only reduced by ~10% in Rab4a-depleted cells; whilst there was a ~30% decrease in tubule length in Rab11a-depleted cells (Figure 7E). These findings suggested key differences in the mechanism of Rab GTPase action on VEGFR2 recycling linked to long-term cellular responses.

**Figure 8:**
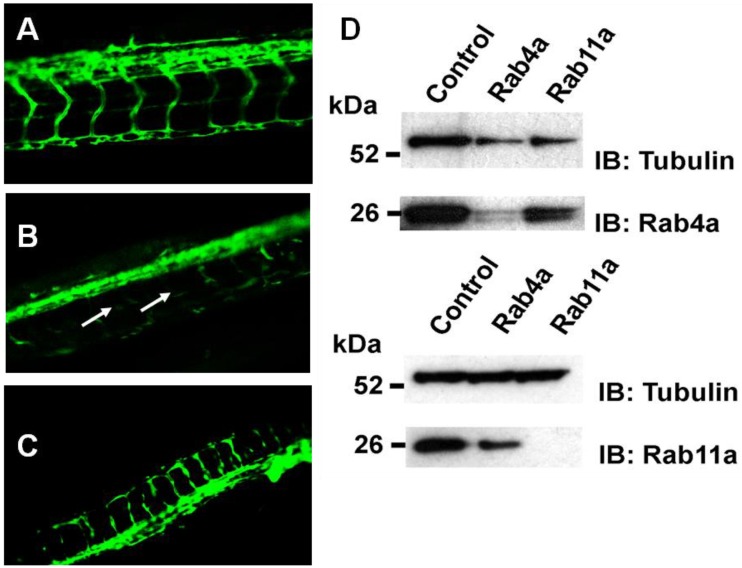
Rab4a regulates vascular development in transgenic zebrafish. Fluorescence microscopy on representative (**A**) control, (**B**) Rab4a-depleted or (**C**) Rab11a-depleted zebrafish transgenic lines (morphants) expressing Fli1:EGFP. Arrows in panel B denote defects in blood vessel formation in Rab4a-depleted animals. (**D**) Immunoblotting of control, Rab4a- or Rab11a-depleted transgenic zebrafish extracts.

To address whether Rab4a and Rab11a activity is important for blood vessel growth and angiogenesis *in vivo*, morpholino-mediated knockdown of these Rab GTPases was carried out in transgenic *Fli1-GFP* zebrafish embryos, where GFP expression is restricted to vascular endothelium in the developing embryo [20,35]. Control scrambled, Rab4a- or Rab11a-specific morpholino antisense oligonucleotides were used to target Rab4a or Rab11a gene expression, and vascular development was analysed (Figure 8). 

In controls (Figure 8A), a well-defined vascular system was evident with intersegmental vessels (ISV) connecting the major dorsal aorta and dorsal longitudinal anastomotic vessel (DLAV). Specific morpholino oligonucleotide-mediated Rab4a depletion (Rab4a morphants) caused severe impairment of both ISV and DLAV formation, with ISVs often missing or prematurely terminated (Figure 8B). In contrast, although Rab11a morphants displayed marked developmental defects (Figure 8C), including some disruption to the embryonic vasculature, both ISV and DLAV appeared to be better preserved than in Rab4a morphants (Figure 8C). Immunoblotting revealed substantial knockdown in protein levels of either Rab4a or Rab11a using specific morpholino antisense oligonucleotides (Figure 8D). Zebrafish morphology upon depletion of either Rab4a or Rab11a also revealed pleiotropic morphological effects, higher developmental abnormalities and mortalities in comparison to controls (S.P. *et al.*, unpublished findings).

### 3.5. Discussion

Here, we provide evidence that a Rab GTPase-dependent endosome-to-plasma membrane recycling step regulates VEGF-A-dependent intracellular signalling, cell migration, proliferation and angiogenesis. VEGFR2 recycles in both the presence and absence of VEGF-A stimulation. The Rab4a and Rab11a GTPases exhibited differential effects on VEGF-A-stimulated cell proliferation and migration. Rab4a depletion caused a dramatic three-fold increase in the VEGF-A-dependent proliferative response. However, Rab11a depletion did not affect endothelial cell proliferation. Rab4a displayed a modest reduction in VEGF-A-stimulated cell migration, whilst Rab11a depletion affected cell migration mediated by both serum and VEGF-A. A VEGF-A-dependent model of endothelial tubulogenesis showed a requirement for both Rab4a and Rab11a, but regulation by both GTPases appeared more crucial for tubule branching rather than extension of tubule length. This observation could in part explain the discrepancy between increased cell proliferation and decreased cell migration in Rab4a-depleted endothelial cells. Rab4a or Rab11a depletion in a transgenic zebrafish model produced different effects on vascular development, indicating the involvement in VEGF-A-regulated processes. However, it is also possible that such effects on animal development are not directly related to VEGF-VEGFR2 function, but rather, are a consequence of the essential roles of such GTPases in trafficking and the function of other membrane receptors.

Overexpression of Rab4a and Rab11a wild-type, or mutant GTPases, indicated a role for Rab4a in VEGFR2 trafficking from early endosomes to the plasma membrane. Comparison of VEGFR2 trafficking upon overexpression of a constitutively active GTP-bound Rab4a-Q67L mutant (S.P. *et al.,* unpublished findings) showed that different Rab4a mutants can cause a block in activated VEGFR2 trafficking through the endosome-lysosome system. Depletion of Rab4a did not significantly affect VEGFR2 levels or phosphorylation status, although downstream activation of Akt, a serine/threonine protein kinase and master regulator, was prolonged. In contrast, Rab11a depletion did not affect Akt activation. Depletion of Rab4a also perturbed activated VEGFR2 proteolysis, consistent with the requirement for VEGFR2 recycling from early endosome to the plasma membrane following ligand stimulation. Thus, a link exists between endosomal Rab4a and the VEGF-A-dependent response in the endothelium. However, it cannot be discounted that other components of signalling pathways (e.g., MAPK enzymes, PDK1, Akt) that localise to the endocytic pathway may also be disrupted by Rab depletion, thus also modulating VEGF-A-stimulated responses.

It has been previously shown that VEGFR2 undergoes recycling from endosomes to the plasma membrane [6,15,21]. However, Mellor and colleagues postulated that although VEGFR2 showed co-distribution with Rab4a-positive endosomes, they argued that VEGFR2 recycles from peripheral endosomes via a Src-dependent pathway [6]. Ballmer-Hofer and colleagues showed that VEGFR2 recycling via Rab4a- or Rab11a-regulated pathways is dependent on VEGFR2 association with NRP1 [21]. We had previously employed both biochemical and microscopy-based assays to show that VEGFR2 does indeed recycle between the cell surface and endosomes, and this was dependent on VEGFR2 tyrosine kinase activity [15]. This study suggests that Rab4a, but not Rab11a, is required for VEGFR2 recycling. This is at variance with previous findings [21]. However, we do observe significant differences between TfR and VEGFR2 recycling, suggesting that the two receptors share common regulatory features (e.g., Rab4a), but also differences in trafficking from endosomes to the plasma membrane. 

The localisation of VEGFR2 along the endocytic pathways is regulated by different GTPases [2,3]. Our previous work showed that Rab5a regulated VEGFR2 trafficking through early endosomes [36]. VEGFR2 early endosome localization was associated with pro-angiogenic signalling from early endosomes, including ERK1/2 activation and cell migration. However, although Rab7a regulated VEGFR2 trafficking out of late endosomes, retention in this compartment decreased pro-angiogenic signal transduction [36]. These findings suggest that quiescent VEGFR2 is efficiently recycled back to the cell surface via Rab4a-positive endosomes; however, this recycling may also occur through Rab11a-positive endosomes, depending on the association with NRP1 [21]. However, if VEGFR2 undergoes activation, phosphorylation and ubiquitination and these post-translational modifications are not removed in the early endosome, activated VEGFR2 progresses towards late endosomes and eventual degradation in lysosomes; compartments that are positioned close to the nucleus [37].

The different effects noted for unchanged VEGFR2 phosphorylation status *versus* prolonged Akt activation following Rab4a depletion represents a possible differential spatio-temporal regulation of these signalling molecules. A mutant Rab4a does not prevent internalisation of VEGFR2 from the plasma membrane (where phosphorylation is initiated), suggesting that such downstream signalling events may be initiated in internal membrane compartments, a phenomenon which has been previously suggested [13,14]. A VEGFR1- and Rab4a-dependent step is also implicated in regulating endothelial αVβ3 integrin recycling and the pro-angiogenic response [38]. Inhibition or depletion of endocytic regulators, including clathrin, ESCRT or Rab proteins, can also affect VEGFR2 trafficking, processing and downstream signalling [12,14,36]. Expression of dominant-negative Rab4a in epithelial cells also inhibits ligand-stimulated degradation of another receptor tyrosine kinase, EGFR/ErbB1 [39].

VEGFR2 undergoes constitutive endocytosis, but displays a lower internalisation rate than the constitutively endocytosed transferrin receptor (~8% per min); this is still a relatively high level of constitutive endocytosis (S.P. *et al.,* unpublished observations). VEGFR2 can localise to both the plasma membrane and intracellular vesicles in endothelial cells. A large fraction of VEGFR2 resides within early endosomes, whilst the remaining fraction is present at the plasma membrane, concordant with previous studies. VEGFR2-mediated cellular outputs have been observed to be linked to lipid rafts and associated factors, which can also modulate signal transduction, trafficking and recycling in other pathways [40,41,42,43,44]. Increased VEGFR2 endocytosis triggered by VEGF-A could be linked to VEGFR2 exit from plasma membrane caveolae or lipid rafts to facilitate receptor-mediated endocytosis. Although plasma membrane VEGFR2 internalisation increased ~1.5-fold upon VEGF-A stimulation (S.P. *et al.,* unpublished observations), this does not account for increased endosome-to-plasma membrane recycling. Inhibition of endosome-to-plasma membrane recycling using the ionophore, monensin, in quiescent cells resulted in the accumulation of VEGFR2 in a perinuclear compartment and a decrease in plasma membrane VEGFR2, when new protein synthesis was also inhibited. This suggests that VEGFR2 is continually recycling through the endosome-plasma membrane system, even in the absence of the VEGF-A ligand.

In contrast to VEGFR2, other transmembrane receptor kinases, such as TGF-β receptor Type I and Type II transmembrane proteins, undergo endocytosis, but recycle via a Rab11a-dependent pathway [45,46]. Rab11a GTPase activity is also implicated in regulating β_2_-adrenergic receptor recycling and degradation [32]. However, other G-protein coupled receptors appear to differentially utilize Rab4a and Rab11a recycling pathways for recycling [25,47]. A comparison of two different prostaglandin G-protein-coupled receptors also suggests differential use of Rab4a *versus* Rab11a recycling pathways [47]. There is also likely to be some overlap between these two routes, as transferrin receptor endosome-plasma membrane recycling is dependent on both Rab4a and Rab11a. One possibility is that membrane proteins are segregated into ‘fast’ or ‘slow’ recycling routes from endosomes depending on the physiological context.

## 4. Conclusions

Members of the Rab family of small GTPases, such as Rab4a and Rab11a, are notable regulators of receptor recycling from endosomes [5,48]. Early and recycling endosomes resemble membrane mosaics containing different regulatory proteins, including Rab GTPases [29,30,49]. Divalent Rab effector proteins further regulate the specificity of protein-protein and protein-lipid interactions within these specialized membrane domains [30]. Interestingly, the dependence of VEGFR2 recycling on Rab4a has similarities to the GLUT4 glucose transporter, which is stored in intracellular vesicles and undergoes insulin- and Rab4a-dependent trafficking to the plasma membrane [50,51,52,53,54]. Rab4a regulates the rapid recycling pathway of the transferrin receptor [26,55] and a G-protein-coupled receptor, cannabinoid receptor CB1 [56]. In contrast, Rab11a is located to perinuclear recycling endosomes and regulates a secondary slow recycling pathway for the transferrin receptor [27,28,29,30] and other G-protein-coupled receptors [25,47,57,58,59]. Our studies have thus demonstrated an essential role for endosome-to-plasma membrane recycling dependence on Rab GTPases. Although Rab4a shows the most involvement in VEGFR2 trafficking and regulation of the pro-angiogenic response, Rab11a appears to also have subtle effects. Our findings thus also suggest that these closely related pathways are highly interlinked, and membrane flux changes in one pathway likely affect the other, with differing consequences for animal physiology.

## Abbreviations

EEA1: early endosome antigen 1
EGFR: epidermal growth factor receptor
eNOS: endothelial nitric oxide synthase
ERK: extracellular signal-regulated kinase
ESCRT: endosomal sorting complex required for transport
FACS: fluorescence-activated cell sorting
GDP: guanosine diphosphate
GTP: guanosine triphosphate
HUVEC: human umbilical vein endothelial cell
PLC: phospholipase C
VEGF-A: vascular endothelial growth factor A
VEGFR: vascular endothelial growth factor receptor
